# IL‐17RA/RC blockade modulates the fibrinolytic system and MMP activity in bleomycin‐induced pulmonary fibrosis in male mice

**DOI:** 10.14814/phy2.70913

**Published:** 2026-06-15

**Authors:** Rakshitha Charavu, Jeena Thrikkandiyoor Madambath, Akarsha B. Jain, Yashodhar P. Bhandary

**Affiliations:** ^1^ Cell Biology & Molecular Genetics Division, Yenepoya Research Centre Yenepoya Deemed to Be University Mangalore Karnataka India; ^2^ Cell and Molecular Biology The University of Texas Health Science Centre San Antonio Texas USA; ^3^ Specialised Research Unit, Yenepoya Medical College & Hospital Yenepoya Deemed to be University Mangalore Karnataka India

**Keywords:** IL‐17A receptors, MMPs, NF‐κB, PAI‐1, pulmonary fibrosis, uPA

## Abstract

Idiopathic pulmonary fibrosis (IPF) is a fatal interstitial lung disease with excessive accumulation of extracellular matrix (ECM) components, which leads to disrupted lung architecture and respiratory failure. Interleukin (IL)‐17 signaling induces fibrosis through inflammation; however, specific roles of its receptors IL‐1RA and IL‐17RC in fibrinolysis and ECM remodeling remain unclear. We hypothesized that inhibition of either IL‐17RA or IL‐17RC could reduce fibrosis by regulating MMP expression and function. In this study, a Bleomycin (BLM)‐induced murine model of pulmonary fibrosis was used to study the effect of neutralization of IL‐17RA and IL‐17RC on ECM remodeling, MMPs, and the fibrinolytic system. Fibrotic lungs showed increased levels of MMP‐2 and MMP‐9, corresponding with elevated expression of fibronectin and collagen. Neutralization of either IL‐17RA or IL‐17RC significantly reduced the expression of MMPs, activation of NF‐κB, and restored normal fibrinolytic activity by upregulating uPA and downregulating PAI‐1 levels.

## 1 BACKGROUND

Idiopathic pulmonary fibrosis (IPF) is a severe form of interstitial lung disease with low life expectancy, a high mortality rate, and limited therapeutic options. IPF is characterized by excessive accumulation of extracellular matrix (ECM) components, which leads to disrupted lung architecture and respiratory failure (Glassberg, 2019). Although the precise etiology of IPF is unknown, studies suggest that a complex interplay of chronic inflammation, fibroblast activation, recurrent epithelial cell damage, and abnormal ECM remodeling is a key feature of disease progression (Chambers & Mercer, 2015; Raghu et al., 2011; Wynn, 2011; Wynn & Ramalingam, 2012).

Matrix metalloproteinases (MMPs) belonging to the zinc‐dependent endopeptidases family are important regulators of ECM remodeling in IPF. MMPs degrade various ECM components and regulate tissue homeostasis (Giannandrea & Parks, 2014; Ra & Parks, 2007). Alterations in MMPs and their tissue inhibitors (TIMPs) lead to excessive ECM deposition (Mei et al., 2022). Dysregulated expression of MMP‐2 and MMP‐9 has been found in BLM‐induced pulmonary fibrosis and in patients diagnosed with IPF (Bormann et al., 2022; Pardo et al., 2016). MMP‐9 is released by inflammatory cells and plays a role in inflammation‐driven tissue remodeling. Epithelial, endothelial, and fibroblast cells produce MMP‐2, which is associated with tissue remodeling and abnormal collagen deposition (Lagente et al., 2005).

In addition to MMPs, the fibrinolytic system contributes to ECM remodeling. Dysregulation of the fibrinolytic components urokinase‐type plasminogen activator (uPA), its receptor (uPAR), and plasminogen activator inhibitor‐1 (PAI‐1) leads to excessive ECM deposition in the lung (Shetty et al., 2008). Apart from the lung, dysregulated fibrinolytic activity is observed in nonalcoholic fatty liver disease (NAFLD) (Eriksen et al., 2022), hepatic cirrhosis (Ogston et al., 1971), renal fibrosis, and glomerulosclerosis (Ma & Fogo, 2009).

Multiple pro‐inflammatory immune cells, including macrophages, neutrophils, and T helper 17 (Th17) cells, have been reported to significantly contribute to pulmonary fibrosis (Wynn & Ramalingam, 2012). Studies have highlighted the role of interleukin (IL)‐17A, a pro‐inflammatory cytokine, produced by Th17 cells, which regulates immune responses and fibrosis‐promoting mechanisms, including tissue repair, the inflammatory response, ECM deposition, and epithelial‐mesenchymal transition (EMT) (Chen & O'Shea, 2008). IL‐17A is known to regulate the recruitment of neutrophils, amplify inflammation, and stimulate the production of various profibrotic mediators and chemokines (Shaikh et al., 2019; Weng et al., 2020). It has been previously shown that IL‐17A contributes to fibrosis through the recruitment of inflammatory cells, the activation of fibroblasts, and ECM deposition in BLM‐induced fibrosis models. IL‐17A signaling enhances the expression of MMPs through the activation of the NF‐κB pathway, which contributes to abnormal ECM remodeling, deposition of collagen, fibronectin, and fibrosis progression (Suyama et al., 2022; Zhang et al., 2021) in several organs such as skin (Speeckaert et al., 2016), liver (Franco et al., 2021), lung (Zhang et al., 2019), kidney (Ge et al., 2014; Ramani et al., 2018; Sun et al., 2018) and heart (Cortez et al., 2007). Multiple studies have reported involvement of IL‐17A signaling in the development of atherosclerotic lesions (Danzaki et al., 2012) and in atherosclerotic effects in ApoE−/− mice (Butcher et al., 2016; Ng et al., 2011; Smith et al., 2010).

Despite growing evidence linking IL‐17A to IPF, the specific role of IL‐17A receptors, IL‐17RA and IL‐17RC, in ECM remodeling and the development of fibrosis remains poorly understood. In this study, we aimed to evaluate the role of IL‐17RA and IL‐17RC in ECM remodeling, MMP‐2 and MMP‐9 expression, and the therapeutic potential of neutralizing IL‐17RA or IL‐17RC in the BLM‐induced pulmonary fibrosis model.

## 2 MATERIALS AND METHODOLOGY

### 2.1 Drugs

Bleomycin (NEON Laboratories Limited, India), IL‐17RA neutralizing antibody (Krishgen biosystems, India, Catalogue number: 10895‐R004), IL‐17RC neutralizing antibody (R&D systems, USA, Catalogue no. AF2270), Ketamine (NEON Laboratories Limited, India), Hematoxylin (Himedia, India, Catalogue no. TC259), Acid fuchsin (Sigma Aldrich, USA, Catalogue no. HT151), Aniline blue (Sigma Aldrich, USA, Catalogue no. B8563), Bradford (Himedia, India, Catalogue No. ML106‐500ML), PVDF (Millipore Sigma, Germany, Catalogue no. IPVH00010), ECL kit (Bio‐RAD, USA, Catalogue no. 1705061) were used in this study.

### 2.2 Antibodies

Primary antibodies used include MMP‐2 (R&D systems, USA, Catalogue no. MAB902‐SP), MMP‐9 (R&D systems, USA, Catalogue no. AF909), uPA (R&D systems, USA, Catalogue no. AF1310‐SP), PAI‐1 (R&D systems, USA, Catalogue no. AF1786‐SP), Fibronectin (R&D systems, USA, Catalogue no. MAB1918‐SP), NF‐κB (Abclonal, USA, Catalogue no. A2547), IL‐6 (R&D systems, USA, Catalogue no. MAB406‐SP), IL‐8 (R&D systems, USA, Catalogue no. MAB208‐SP), β‐actin (R&D systems, USA, Catalogue no. MAB8969).

Secondary antibodies included Alexa Fluor 488 anti‐rabbit (Invitrogen, USA, Catalogue no. A31573), Alexa Fluor 488 anti‐goat (Invitrogen, USA, Catalogue no. A11055), and Alexa Fluor 488 anti‐mouse (Invitrogen, USA, Catalogue no. A‐21202), anti‐mouse horseradish peroxidase‐conjugated (HRP) (CST, USA, Catalogue no. 7076P2), and anti‐goat HRP‐conjugated (R&D systems, USA, Catalogue no. HAF019).

### 2.3 Animals and experimental design

C57BL/6 male mice, aged 6 to 8 weeks and weighing 25 ± 5 g, were obtained from Adita Biosys Private Limited, Tumkuru, India (Reference number: SL/DC/086B/24–25). The mice were housed in individually ventilated cages (IVC) (3–4 mice per cage) with sterile corn cob as bedding material. Animals were maintained in controlled environmental conditions (temperature 22°C ± 2°C, 12 h light/12 h dark cycle and humidity 50%–60%) with unrestricted access to food and water.

All experimental procedures were conducted in accordance with CPCSEA regulations and approved by the Institutional Animal Ethics Committee at Yenepoya University (approval number: YU/IAEC/P12/2024).

Animals were randomly divided into 7 groups (*n* = 6 per group): Control, Bleomycin (BLM), IL‐17RA neutralizing antibody, IL‐17RC neutralizing antibody, BLM + IL‐17RA neutralizing antibody, BLM + IL‐17RC neutralizing antibody. An additional group received treatment with both IL‐17RA + IL‐17RC neutralizing antibodies to assess potential additive or synergistic effects of dual receptor blockade. Animals were sacrificed at 14 and 21 days following BLM administration.

### 2.4 BLM‐induced fibrosis and treatment with IL‐17RA and IL‐17RC neutralizing antibodies

C57BL/6 mice were categorized into seven groups (*n* = 6 per group): control (saline), BLM (2 mg/kg body weight), IL‐17RA neutralizing antibody (1 μg), IL‐17RC neutralizing antibody (1 μg per mouse) (Ding et al., 2017; Willis et al., 2015; Ye et al., 2019), BLM (2 mg/kg body weight) with IL‐17RA neutralizing antibody (1 μg per mouse), BLM (2 mg/kg body weight) with IL‐17RC neutralizing antibody (1 μg per mouse), and BLM (2 mg/kg body weight) with both IL‐17RA neutralizing antibody (1 μg per mouse) and IL‐17RC neutralizing antibody (1 μg per mouse).

Pulmonary fibrosis was induced by intranasal administration of BLM (2 mg/kg body weight) on Day 0. BLM stock solution (60 μg/μL) was prepared in sterile water and diluted 1:10 to obtain a working concentration of 6 μg/μL.

**FIGURE 1:**
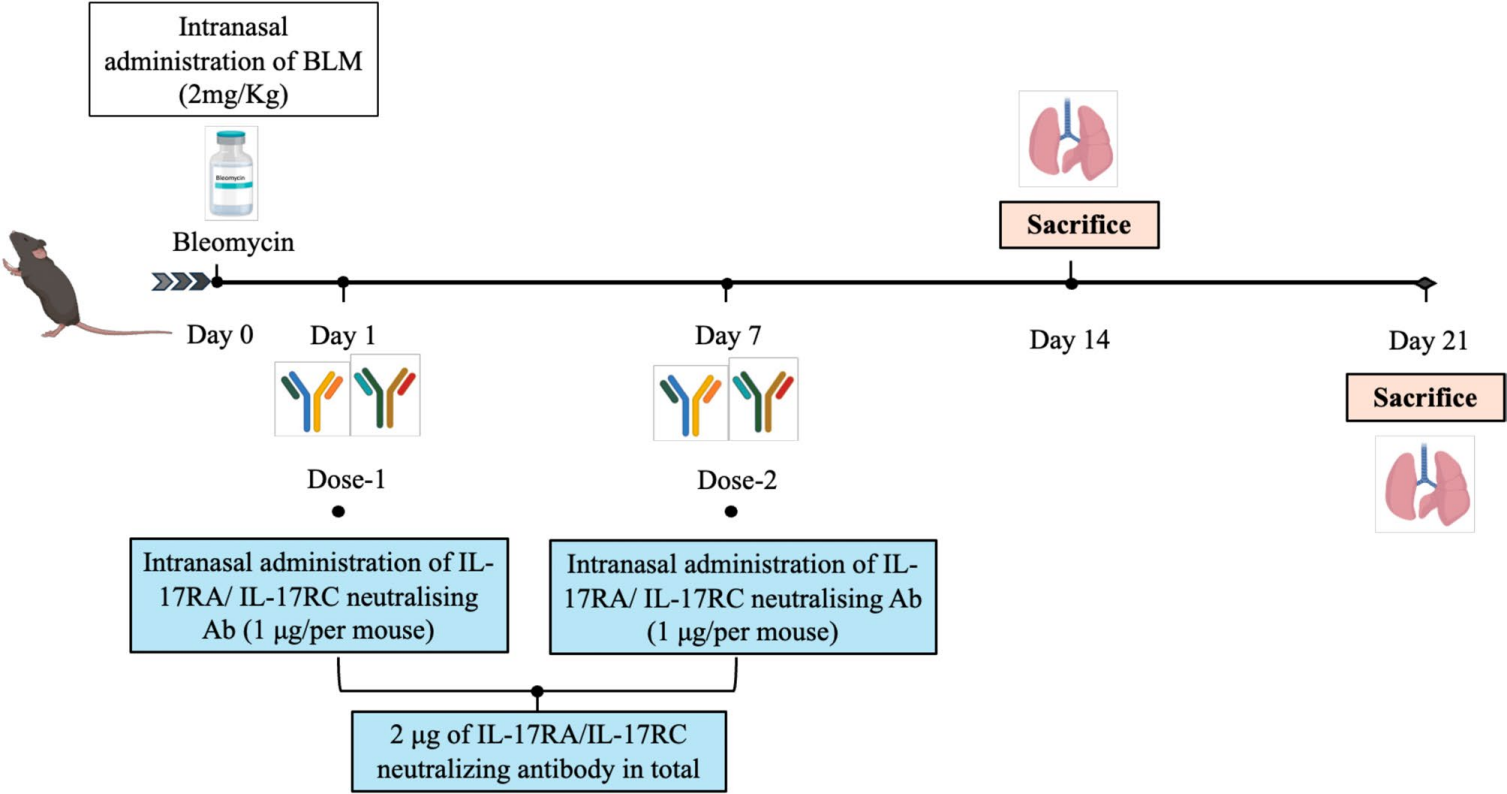
Schematic representation of experimental design for IL‐17RA/RC neutralization in BLM‐induced pulmonary fibrosis.

Mice were anesthetized with ketamine (0.02–0.04 mg) and administered BLM. The first dose of IL‐17RA (1 μg per mouse) and/or IL‐17RC (1 μg per mouse) neutralizing antibodies was administered intranasally 24 h after BLM exposure. A second dose of neutralizing antibodies was given on day 7 post‐BLM administration. Mice were euthanized with a higher dose of ketamine (100 mg/kg body weight) at 14 and 21 days following BLM exposure. Lung tissues were collected for further analysis (Figure 1).

### 2.5 Histopathological analysis

Mice were euthanised at 14 and 21 days following BLM exposure. Whole lungs were excised and fixed in 10% formalin. Tissues were dehydrated using different gradients of ethanol, embedded in paraffin using a Paraffin embedding module (Leica, model: EC1150H), and sectioned at a thickness of 3 μm using a microtome (Leica, model: RM2245). Sections were mounted on glass slides.

### 2.6 Hematoxylin and eosin staining

Lung sections were deparaffinized using xylene and rehydrated using graded ethanol (100%, 95%), followed by water. Sections were stained with hematoxylin for 3 min, rinsed, and differentiated in 1% acid alcohol. The sections were then stained with Scott's bluing reagent for 1 min and counterstained with 0.25% eosin for 1 min.

The sections were rinsed and dehydrated using 95% ethanol, 100% ethanol, and xylene, mounted using DPX and examined under a light microscope (Magcam DC5 5.1MP CMOS sensor, 20x). Fibrotic changes were assessed using the Ashcroft scoring system. Five nonoverlapping fields per section were assessed under 20x magnification.

### 2.7 Masson's trichrome assay

Lung sections (3 μm thickness) were deparaffinized using xylene and subsequently rehydrated with various concentrations of alcohol. The sections were stained with Weigert's Iron Hematoxylin for 5 min, followed by Biebrich Scarlet‐Acid Fuchsin for 5 min. After differentiation in 1% Phosphomolybdic‐Phosphotungstic Acid for 2 min, sections were stained with aniline blue for 5 min. Sections were then dehydrated, mounted with DPX, and visualized under a light microscope (Magcam DC5 5.1MP CMOS sensor, 20X). Fibrosis was evaluated using Ashcroft scoring. Five nonoverlapping fields per section and five sections per mouse were analyzed.

### 2.8 Immunofluorescence analysis

Lung sections (3 μm) were deparaffinized using xylene and graded ethanol series. Sections were permeabilised with a 0.4% Triton X‐100 for 5 min and blocked using 5% FBS for 1 h at room temperature. Sections were treated with primary antibody (1:200 dilution) overnight at 4°C, followed by incubation with Alexa Fluor secondary antibody (1:2000 dilution) for 1 h in the dark. The images were captured using ZOE imager (Bio‐RAD, USA).

### 2.9 Protein isolation

Mouse lungs were homogenized in 4% SDS protein extraction buffer using a Dounce homogenizer. The lung homogenate was subjected to heating at 95°C for 10 min and subsequently centrifuged at 10,000 rpm for 5 min. The supernatant was harvested and preserved at −80°C. The concentration of the purified protein samples was measured with the Bradford assay.

### 2.10 Western blot

SDS‐PAGE was conducted using 10% gels. The proteins were electroblotted onto the PVDF membrane at 4°C for 2 h. Membranes were blocked with 5% skim milk for 1 h and incubated with the primary antibodies (1:1000 dilution) overnight at 4°C. Membranes were then incubated with HRP‐conjugated secondary antibodies (1:3000 dilution) for 1 h at room temperature. Protein bands were visualized using an enhanced chemiluminescence detection system (Bio‐Rad, USA).

### 2.11 Statistical analysis

The outcomes of our study were evaluated using GraphPad Prism (8.01). The data are presented as mean values ± standard deviation (SD). Statistical comparisons were performed using one‐way analysis of variance (ANOVA) followed by Sidak's multiple comparison test. Statistical significances are defined by *p < 0.05, **p < 0.01, and ***p < 0.001. Exact *p* values were calculated and are mentioned in the figures.

## 3 RESULTS

### 3.1 Treatment with IL‐17RA or IL‐17RC neutralizing antibodies resolves lung inflammation

**FIGURE 2:**
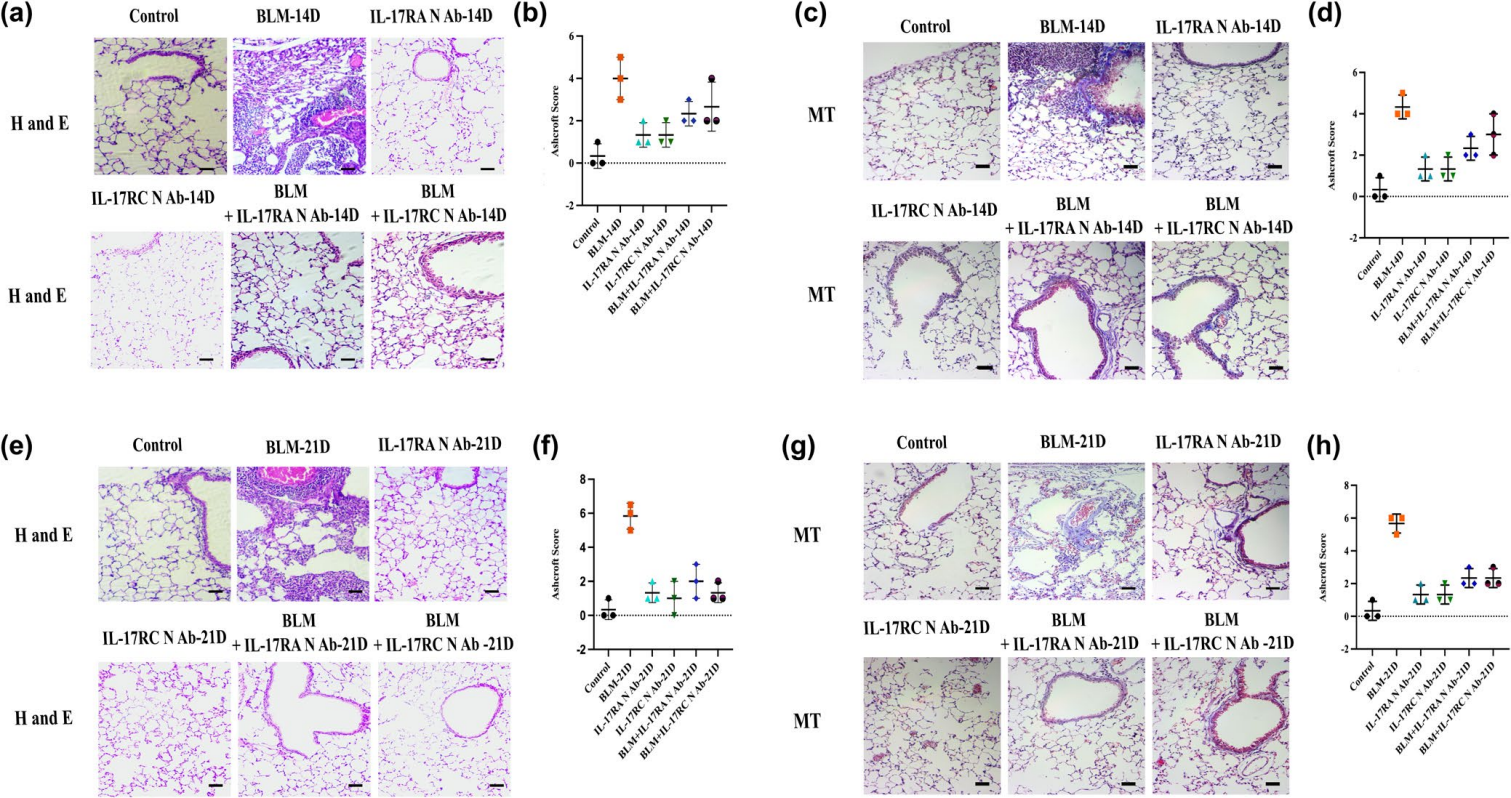
Histological analysis and Ashcroft scoring reveal attenuation of fibrosis on treatment with IL‐17RA or IL‐17RC neutralizing antibodies in BLM‐induced pulmonary fibrosis: Hematoxylin and eosin (H and E) staining of (a) Lung morphology on day 14 post‐BLM, (b) Ashcroft scoring corresponding to H and E staining performed at 14th day, (c) Lung morphology on day 21 post‐BLM, (d) corresponding Ashcroft scoring at 21st day. (e) Masson Trichrome (MT) staining at 14 day posttreatment (f) the representative Ashcroft scoring at 14th day, (g) MT staining at 21‐day posttreatment (f) the representative Ashcroft scoring at 21st day.

Histological analysis of mouse lungs collected on days 14 and 21 post‐BLM administration showed disrupted alveolar architecture and alveolar wall thickening, indicating extensive lung damage in mice exposed to BLM. In contrast, mice treated with IL‐17RA or IL‐17RC neutralizing antibodies showed a notable reduction in inflammation and partial restoration of alveolar architecture at both time points (Figure 2a,c). Consistent with these observations, Ashcroft scores were significantly higher in mice exposed to BLM than in controls, whereas mice treated with IL‐17RA or IL‐17RC neutralizing antibodies reduced the scores, reflecting improved lung architecture (Figure 2b,d).

### 3.2 IL‐17RA or IL‐17RC neutralization restores normal fibrinolytic activity in vivo

**FIGURE 3:**
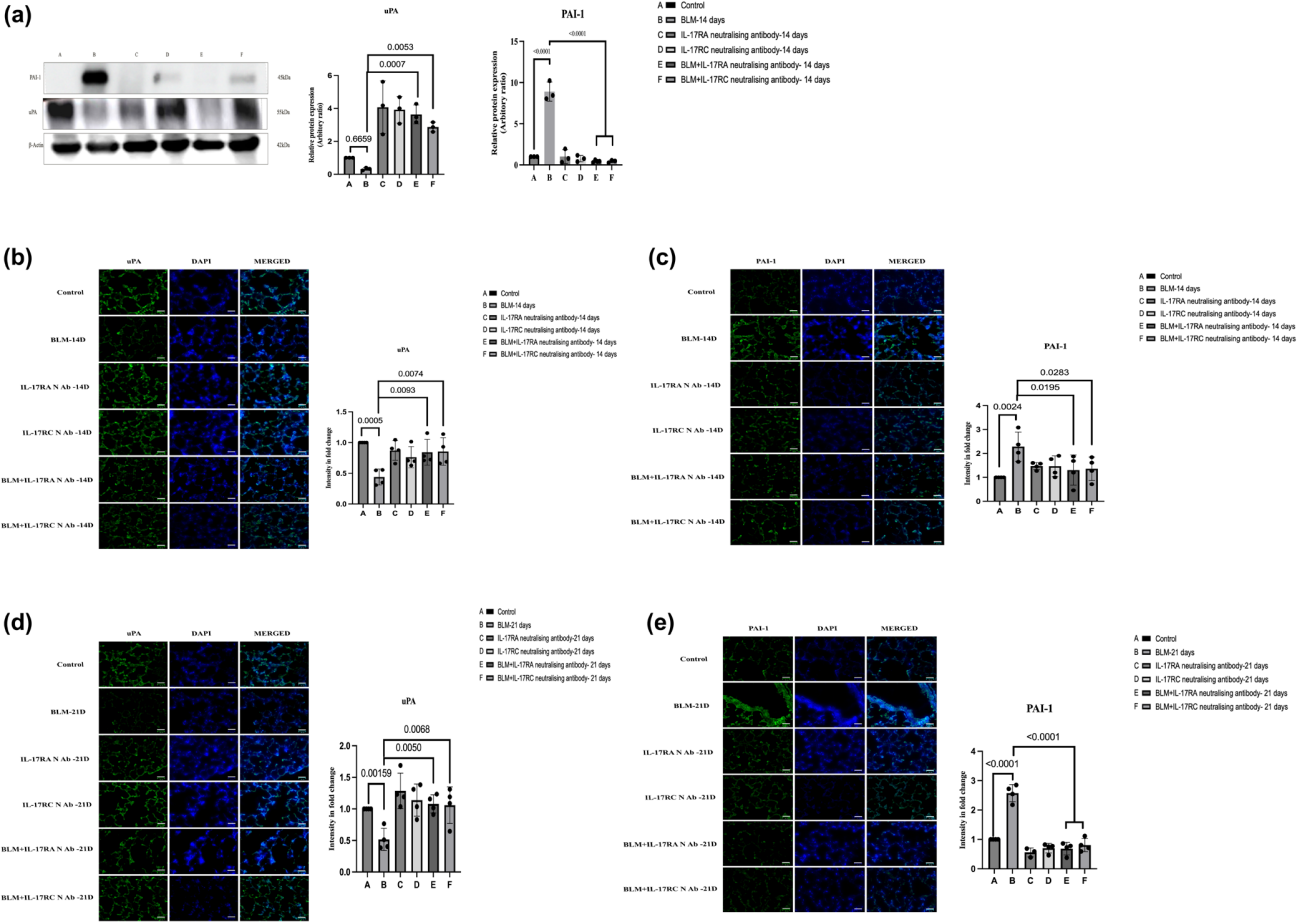
Treatment with IL‐17RA or IL‐17RC neutralizing antibodies restores fibrinolytic system in BLM‐induced lung fibrosis: (a) Representative blots and densiometric analysis of uPA and PAI‐1 at 14th day postadministration of BLM. The immunofluorescence images with respective graph of (b) uPA at 14 day post‐BLM treatment, (c) PAI‐1 at 14 days post‐BLM treatment, (d) uPA at 21 days post‐BLM administration, (e) PAI‐1 at 21 days post‐BLM treatment. Scale bar‐25 μm. Data were analyzed using One‐way ANOVA and Sidak's multiple comparison test. Data are shown as mean ± SD. Fu‐l length blots with molecular weight markers are provided in the Figure S1.

Western blot analysis revealed an increase in the expression of PAI‐1 and decreased expression of uPA in BLM‐exposed groups at day 14. Treatment with IL‐17RA or IL‐17RC neutralizing antibodies upregulated the expression of uPA and downregulated the expression of PAI‐1 (Figure 3a). Further, immunostaining results showed decreased expression of PAI‐1 and increased expression of uPA in mice treated with IL‐17RA or IL‐17RC neutralizing antibodies 14 days after BLM administration, suggesting that treatment with IL‐17RA or IL‐17RC neutralizing antibodies can restore normal fibrinolytic activity (Figure 3b,c). Immunostaining of uPA and PAI‐1 showed increased expression of uPA and decreased expression of PAI‐1 21 days following BLM administration (Figure 3d,e). These results suggest that treatment with either IL‐17RA or IL‐17RC neutralizing antibodies could restore normal fibrinolytic function in BLM‐induced pulmonary fibrosis.

### 3.3 IL‐17RA or IL‐17RC neutralizing antibodies reduce ECM deposition in BLM induced‐pulmonary fibrosis in vivo

To evaluate collagen deposition, Masson trichrome staining was performed. Results revealed extensive collagen deposition, indicating fibrosis progression in lung sections of mice exposed to BLM for both 14 and 21 days. Treatment with IL‐17RA or IL‐17RC neutralizing antibodies in mice exposed to BLM (14 and 21 days) reduced collagen deposition and preserved normal lung architecture compared to mice exposed to only BLM (Figure 2b).

**FIGURE 4:**
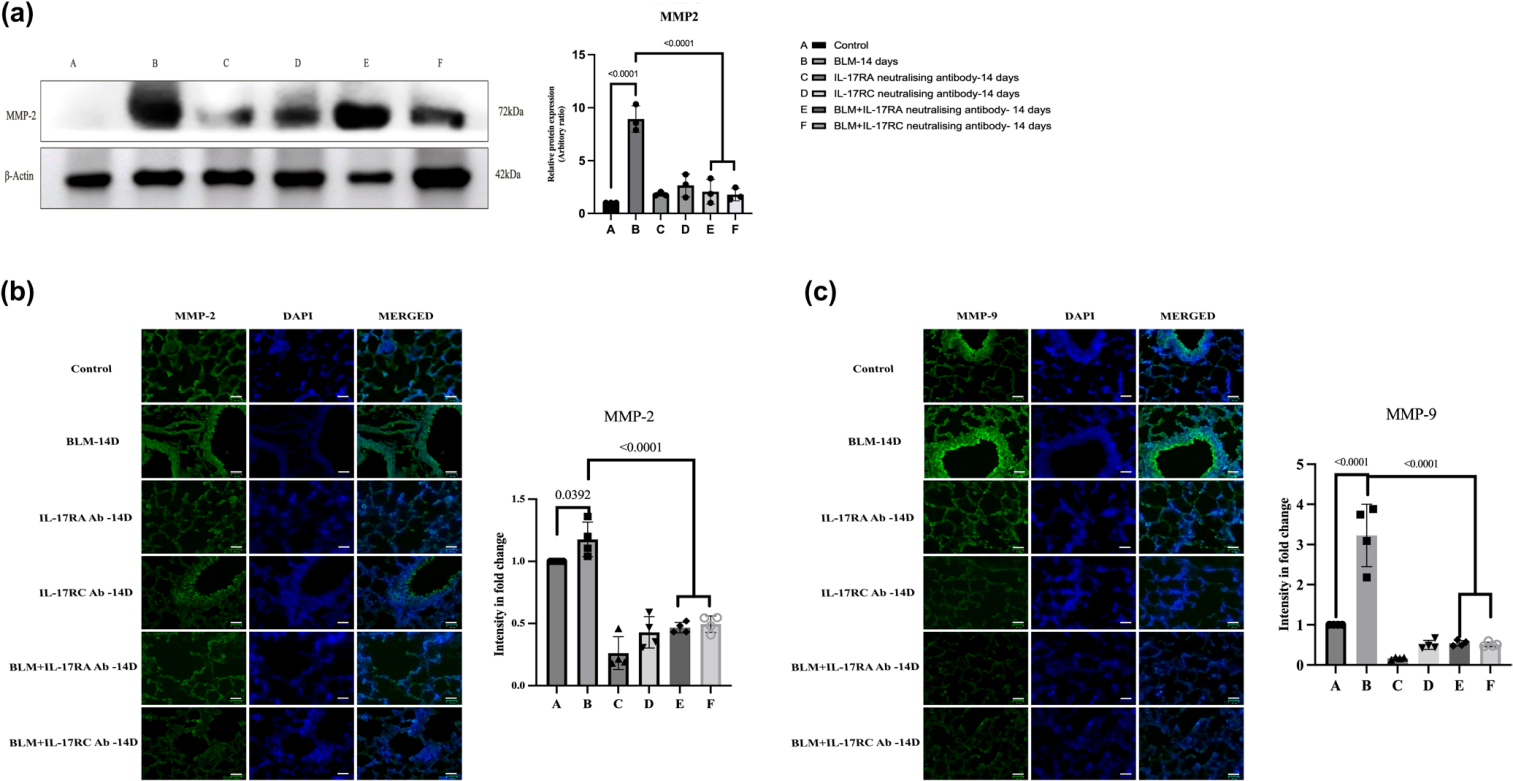
Treatment with IL‐17RA or IL‐17RC neutralizing antibodies reduces MMP‐2 and MMP‐9 expression and restores fibrinolytic system in BLM‐induced lung fibrosis: (A) Representative blots and densiometric analysis of MMP‐2 at 14th day post‐BLM administration, β‐Actin was used as loading control. The immunofluorescence images with representative graphs of (b) MMP‐2 at 14 days post‐BLM administration, (c) MMP‐9 at 14 days post‐BLM treatment, Scale bar‐25 μm. Data were analyzed using One‐way ANOVA and Sidak's multiple comparison test. Data are shown as mean ± SD. Full‐length blots with molecular weight markers are provided in the Figure S1.

**FIGURE 5:**
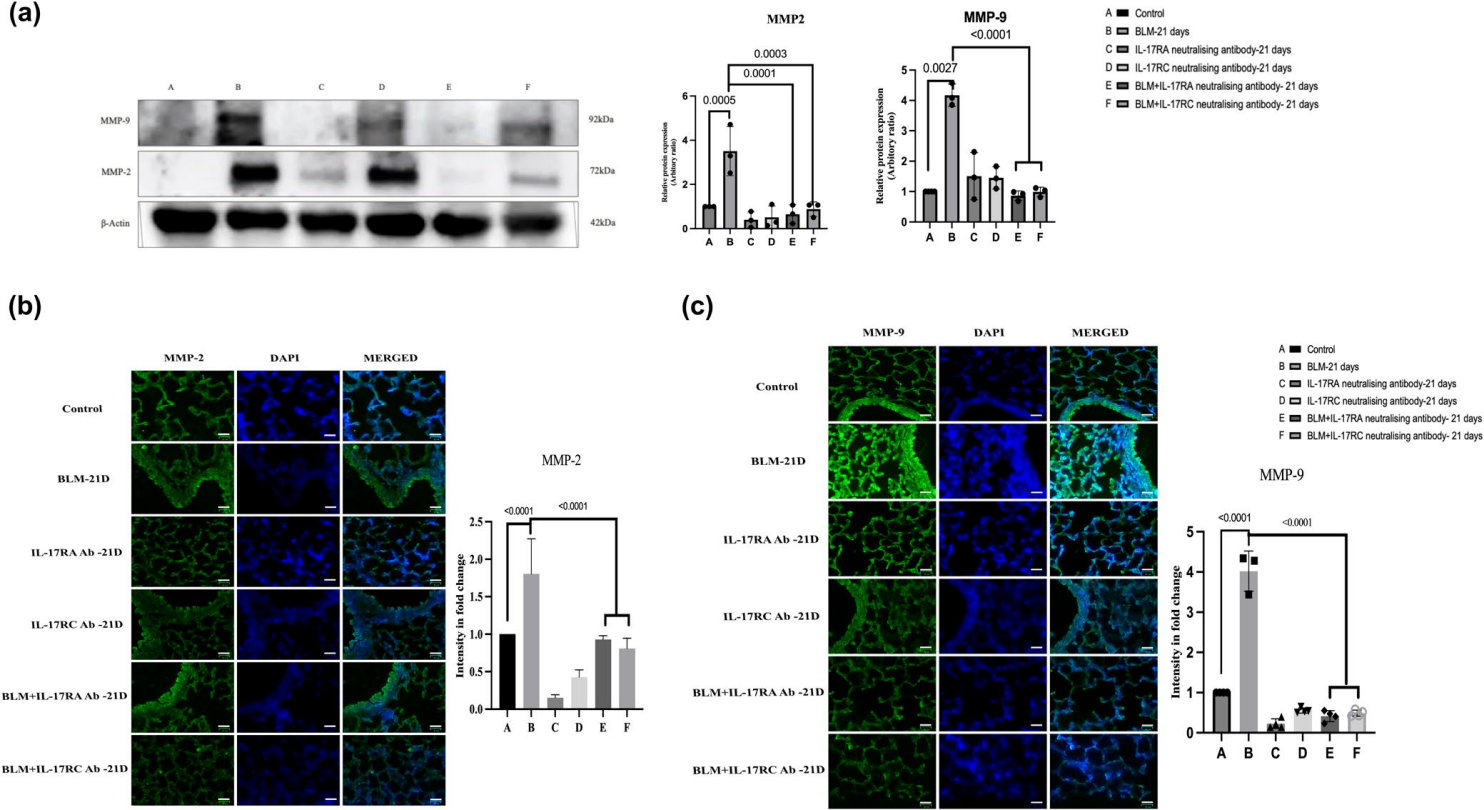
Treatment with IL‐17RA or IL‐17RC neutralizing antibodies reduces MMP‐2 and MMP‐9 expression and restores the fibrinolytic system in BLM‐induced lung fibrosis: (A) Representative blots and densiometric analysis of MMP‐2 and MMP‐9 21 days post‐BLM administration. β‐Actin was used as a loading control. The immunofluorescence images with respective graphs of (b) MMP‐2 at 21 days post‐BLM administration, (c) MMP‐9 at 21 days post‐BLM treatment, Scale bar‐25 μm. Data were analyzed using One‐way ANOVA and Sidak's multiple comparison test. Data are shown as mean ± SD. Full‐length blots with molecular weight markers are provided in the Figure S1.

Western blot results showed marked up‐regulation of MMP‐2 expression at both 14 and 21 days following BLM administration, and increased expression of MMP‐9 was seen 21 days following BLM administration, suggesting accumulation of ECM components. Treatment with IL‐17RA or IL‐17RC neutralizing antibodies significantly reduced the expression of MMP‐2 at both 14 and 21 days postadministration of BLM and the expression of MMP‐9 at 21 days after BLM administration (Figure 4a, Figure 5a).

Further, immunostaining was performed, and the results supported the western blot data. The results showed enhanced expression of MMP‐2 and MMP‐9 at 14 and 21 days postexposure to BLM. On treatment with IL‐17RA or IL‐17RC neutralizing antibodies, expression of MMP‐2 and MMP‐9 was substantially reduced at both 14 (Figure 4b,c) and 21 days (Figure 5b,c).

**FIGURE 6:**
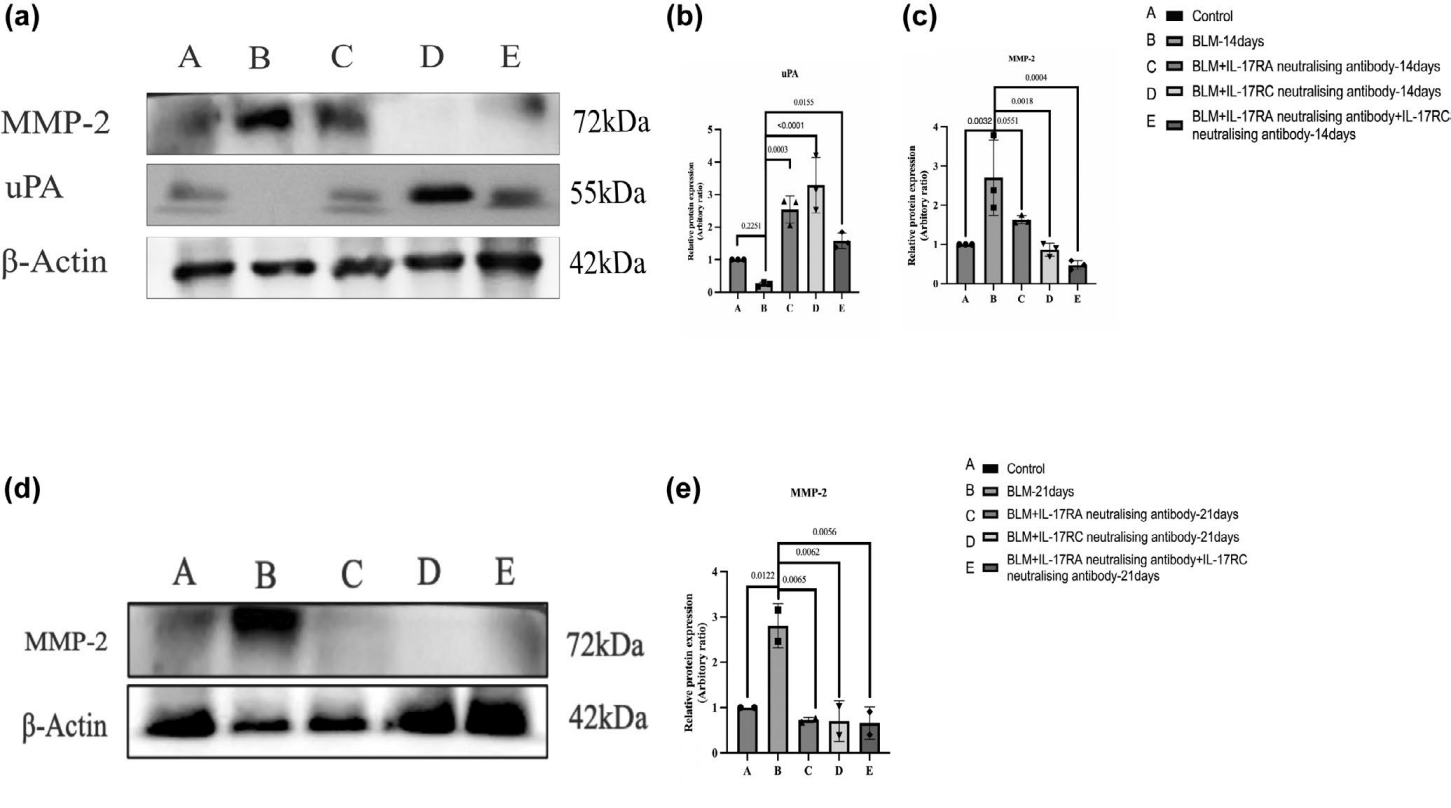
Differential regulation of MMP‐2 and uPA by IL‐17A receptors: (a) Representative western blot image of uPA and MMP‐2, (b) densiometric analysis of uPA and (c) densiometric analysis of MMP‐2 14 days post‐BLM administration, (d) Representative western blot image of MMP‐2, (b) densiometric analysis of MMP‐2 21 days post‐BLM administration. Data were analyzed using One‐way ANOVA and Sidak's multiple comparison test. Data are shown as mean ± SD. Full‐length blots with molecular weight markers are provided in the Figure S1.

To evaluate the comparative effects of IL‐17RA and IL‐17RC on the expression of MMPs and uPA, western blot was performed following treatment with IL‐17RA and IL‐17RC neutralizing antibodies in C57BL/6 mice. Treatment with IL‐17RA neutralizing antibody increased the expression of uPA and moderately decreased the expression of MMP‐2 14 days post‐BLM administration. Treatment with IL‐17RC neutralizing antibody resulted in significant upregulation of uPA and downregulation of MMP‐2 in mice 14 days post‐BLM administration. Combined treatment of both IL‐17RA and IL‐17RC neutralizing antibodies upregulated uPA expression, and there was substantial downregulation of MMP‐2 14 days post‐BLM administration (Figure 6a–c). Treatment with IL‐17RA and IL‐17RC neutralizing antibodies alone post‐BLM administration (21 days) downregulated MMP‐2 levels. Notably, treatment with both IL‐17RA and IL‐17RC neutralizing antibodies led to a trend towards greater reduction of MMP‐2 levels in mice 21 days post‐BLM treatment (Figure 6d,e).

**FIGURE 7:**
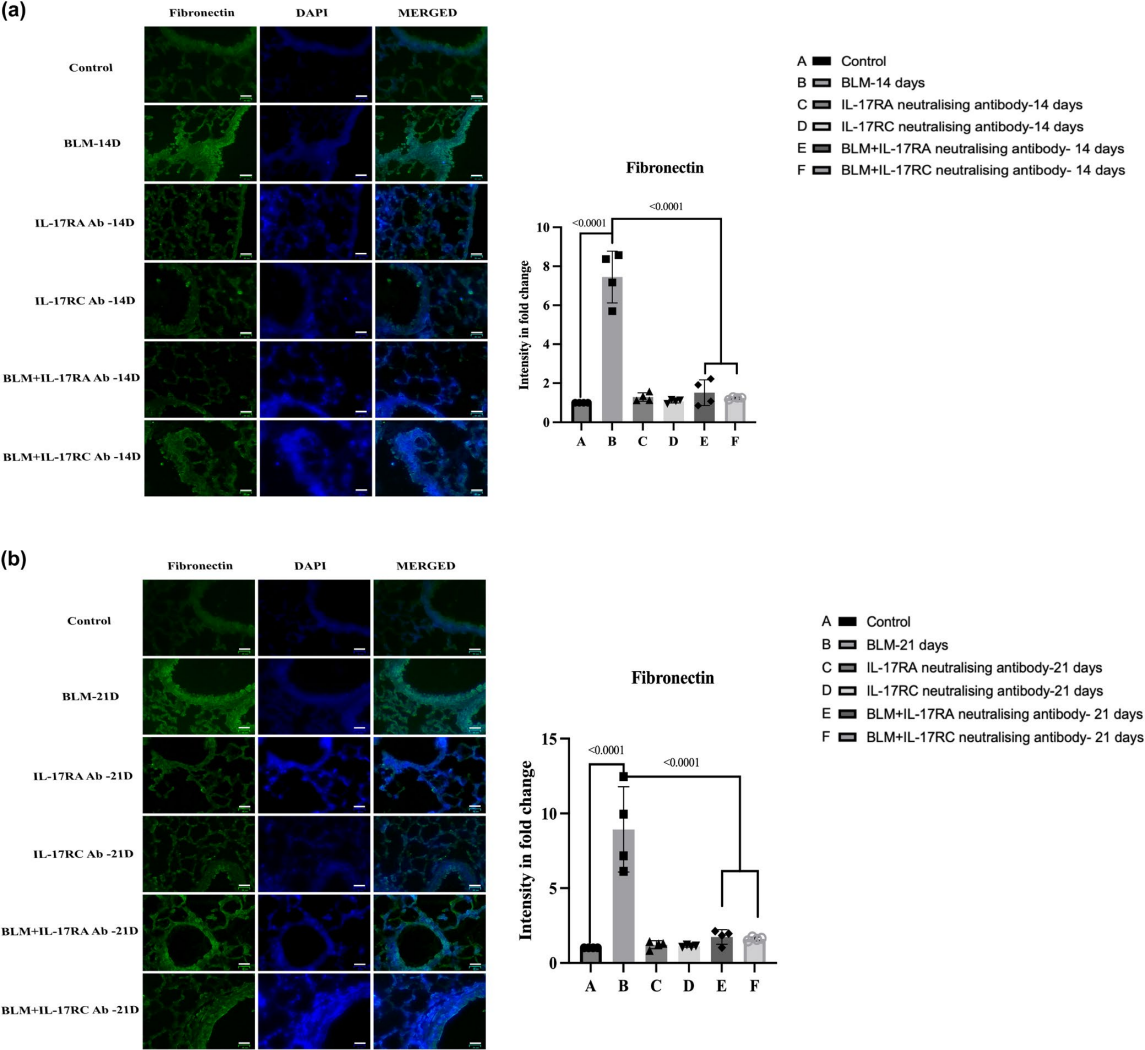
IL‐17RA and IL‐17RC blockade attenuates fibrosis and ECM protein expression in bleomycin‐induced lung injury: Immunofluorescence images of fibronectin at (a) 14 and (b) 21 days post‐BLM treatment. Scale bar: 25 μm. Data were analyzed using One‐way ANOVA and Sidak's multiple comparison test. Data are shown as mean ± SD.

Immunofluorescence staining demonstrated increased fibronectin expression in BLM‐treated lung tissues, signifying excessive ECM buildup. The treatment with IL‐17RA or IL‐17RC neutralizing antibodies significantly reduced fibronectin expression on both days 14 and 21 (Figure 7a,b). These findings suggest that IL‐17RA and IL‐17RC are involved in promoting fibrotic remodeling, and treatment with these antibodies may attenuate the progression of fibrosis.

### 3.4 Treatment with IL‐17RA or IL‐17RC neutralizing antibodies attenuates NF‐κB activation and downstream pro‐inflammatory cytokines in BLM‐induced pulmonary fibrosis

**FIGURE 8:**
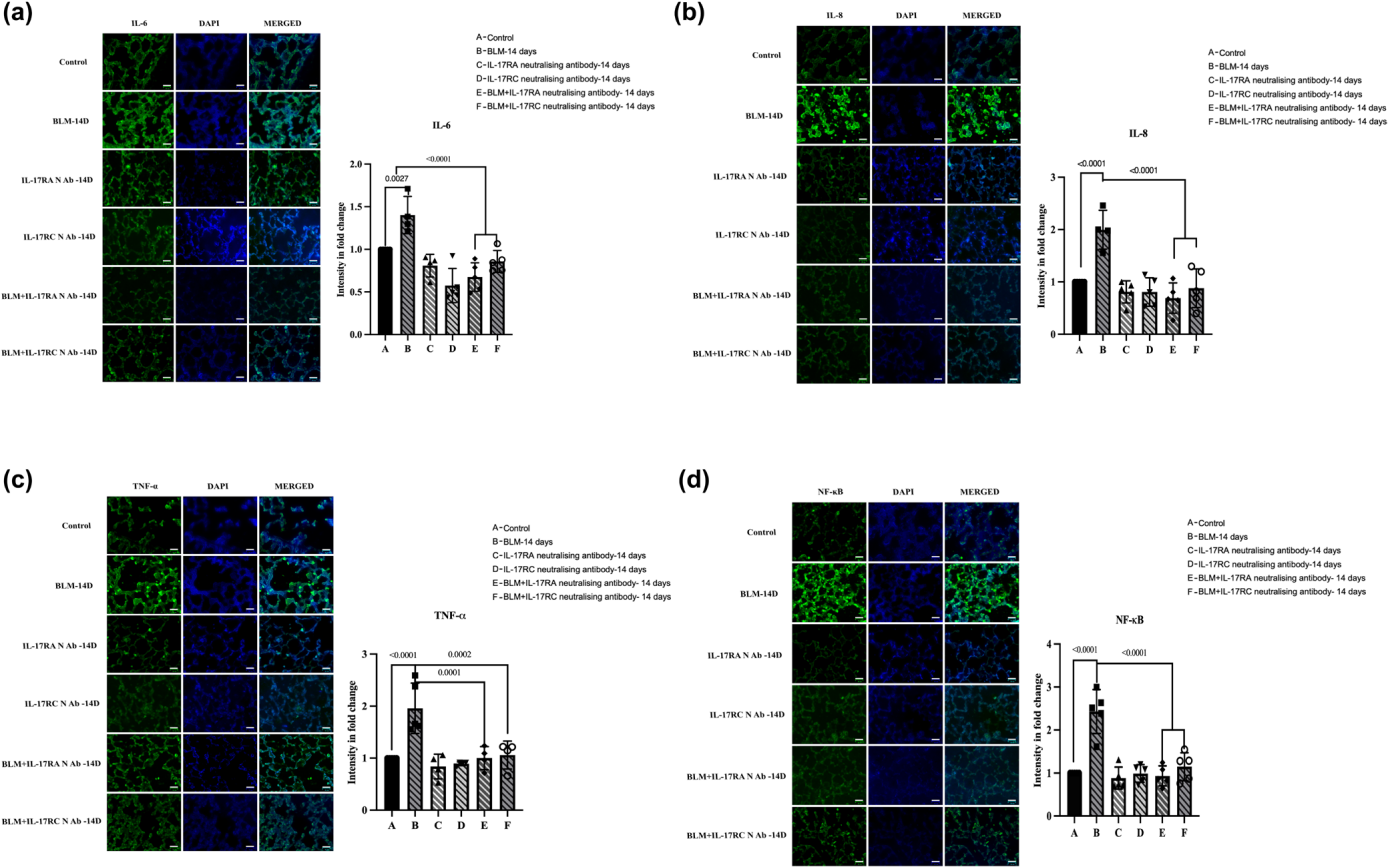
Treatment with IL‐17RA or IL‐17RC neutralizing antibodies attenuates NF‐κB activation and downstream pro‐inflammatory cytokines in BLM‐induced pulmonary fibrosis: Representative immunofluorescence images and corresponding graphs are shown for (a) IL‐6, (b) IL‐8, (c) TNF‐α, and (d) NF‐κB. Scale bar: 25 μm. Data were analyzed using one‐way ANOVA and Sidak's multiple comparison test. Data are shown as mean ± SD.

**FIGURE 9:**
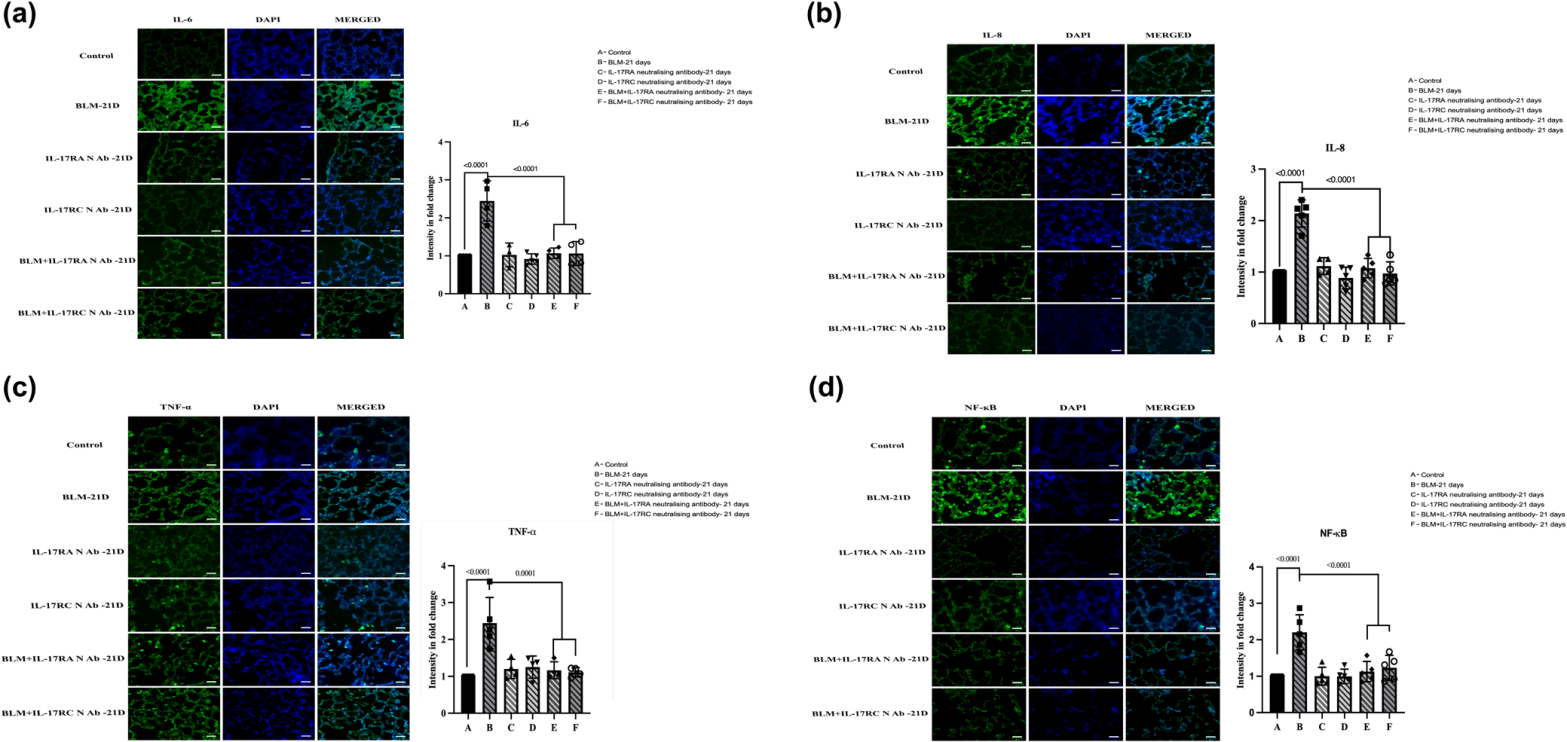
Treatment with IL‐17RA or IL‐17RC neutralizing antibodies attenuates NF‐κB activation and downstream pro‐inflammatory cytokines in BLM‐induced pulmonary fibrosis: Representative immunofluorescence images and corresponding graphs are shown for (a) IL‐6, (b) IL‐8, (c) TNF‐α, and (d) NF‐κB. Scale bar—25 μm. Data were analyzed using One‐way ANOVA and Sidak's multiple comparison test. Data are shown as mean ± SD.

To assess the impact of treatment with IL‐17RA and IL‐17RC neutralizing antibodies on NF‐κB activation, we performed immunostaining of NF‐κB, IL‐6, IL‐8, and TNF‐α. Expression of IL‐6, IL‐8, TNF‐α, and NF‐κB was significantly increased in mouse lungs 14 days post‐BLM administration, indicating activation of NF‐κB. Treatment with either IL‐17RA or IL‐17RC neutralizing antibodies significantly reduced the expression of IL‐6, IL‐8, TNF‐α, and NF‐κB (Figure 8a–d). Further immunostaining of mouse lung sections 21 days post‐BLM treatment showed that treatment with IL‐17RA or IL‐17RC neutralizing antibodies downregulated the expression of NF‐κB and its downstream molecules, IL‐6, IL‐8, and TNF‐α (Figure 9a–d). Treatment with either IL‐17RA or IL‐17RC neutralizing antibodies resulted in a reduction in the expression of NF‐κB and its downstream pro‐inflammatory cytokine, suggesting both receptors contribute similarly to the activation of NF‐κB in the BLM‐induced pulmonary fibrosis model.

## 4 DISCUSSION

In this study, we aimed to investigate how treatment with IL‐17RA and IL‐17RC neutralizing antibodies modulates MMPs and ECM deposition in a murine model of BLM‐induced pulmonary fibrosis. We found that mice exposed to BLM showed substantial alveolar damage, increased collagen and fibronectin deposition, and elevated expression of MMP‐2 and MMP‐9. Ashcroft scores indicated that treatment with either IL‐17RA or IL‐17RC neutralizing antibodies suppresses fibrotic remodeling, activation of fibroblasts, and ECM deposition.

The combined neutralization of IL‐17RA and IL‐17RC receptors was done to evaluate the synergistic effect of dual neutralization compared to individual receptor neutralization. Combined treatment of IL‐17RA and IL‐17RC neutralizing antibodies reduced the expression of MMPs significantly. The difference between individual receptor blockade and combined blockade may be caused by differences in their interactions and downstream signaling. However, the combination treatment group received bleomycin to test its therapeutic potential under fibrosis. These results align with previous studies that reported significantly elevated levels of MMP‐2 and MMP‐9 in both experimental models of lung fibrosis and individuals with IPF (Bormann et al., 2022; McKeown et al., 2009; Mishra et al., 2011; Todd et al., 2020). Elevated expression of MMP‐2 and MMP‐9 levels was reported in the bronchoalveolar lavage fluid and lung homogenates of mice subjected to AdTGF‐β1‐induced fibrosis (Bormann et al., 2022). Interleukin (IL)‐17A is a known pro‐inflammatory cytokine produced by Th17 cells, and it has been associated with neutrophil recruitment and tissue remodeling (Zhang et al., 2019). Studies on the murine model of BLM‐induced lung fibrosis revealed that IL‐17A is involved in multiple fibrotic signaling pathways and promotes deposition of ECM components (Gasse et al., 2011; Lei et al., 2016; Mi et al., 2011; Wilson et al., 2010). Several studies showed that neutralization of IL‐17A delayed the immune response and effectively diminished lung inflammation and fibrosis in many animal models (Chen et al., 2014; Cipolla et al., 2017; Mi et al., 2011).

A similar study on the role of IL‐17A stimulation of hepatic fibrosis shows that the fibrotic liver tissues brought on by cholestatic liver injury have higher expressions of IL‐17A, IL‐17A‐related molecules, and elements of the IL‐17A signaling pathway (Franco et al., 2021; Kartasheva‐Ebertz et al., 2022). Research has pointed to the potential role of IL‐17A in inducing inflammation and fibrotic alteration by activating the CXCL5/CXCR2 signaling axis in a condition called Rosacea, which can lead to fibrosis (Zhang et al., 2024). Furthermore, studies in sterile pericarditis (SP) models have demonstrated that elevated levels of pro‐inflammatory cytokines (IL‐6, IL‐1β, and TGF‐β1) contribute to atrial inflammation and fibrosis. These changes are associated with increased susceptibility to atrial fibrillation (AF), which has been linked to elevated IL‐17A levels (Huang, 2024).

Aligning with previous reports, our study provides evidence supporting the important role of IL‐17A and IL‐17A receptors in the development of pulmonary fibrosis. Treatment with IL‐17RA or IL‐17RC neutralizing antibodies attenuated inflammation. Mice treated with IL‐17RA or IL‐17RC neutralizing antibodies post‐BLM exposure showed preserved lung architecture and reduced infiltration of immune cells. It also reduced deposition of ECM components such as collagen, fibronectin, and modulated the expression of MMP‐2 and MMP‐9 at both 14 and 21 days post‐BLM exposure. This indicates treatment with IL‐17RA or IL‐17RC neutralizing antibodies could reduce ECM deposition by blocking IL‐17A signaling during pulmonary fibrosis.

IL‐17A is known to contribute to the expression of MMPs and pro‐inflammatory cytokines through the NF‐κB pathway (Feng et al., 2019; Suyama et al., 2022; Zhang et al., 2021), which contributes to excess deposition of ECM and fibrosis progression. In this study, we observed that treatment with either IL‐17RA or IL‐17RC neutralizing antibodies could suppress activation of NF‐κB and expression of IL‐6, IL‐8, and TNF‐α. As IL‐17RA serves as a receptor to multiple members of the IL‐17 family, treatment with IL‐17RA neutralizing antibody may exert a broader effect on NF‐κB activation compared to treatment with IL‐17RC neutralizing antibody. TGF‐β has been well‐characterized as an inducer of fibrosis, activating fibroblasts and regulating extracellular matrix production. Additional research involving the interaction between IL‐17R signaling and TGF‐β could provide further understanding regarding the progression of fibrosis. While activation of NF‐κB was decreased after inhibition of IL‐17RA and IL‐17RC, the present study describes an association between IL‐17R signaling and NF‐κB signaling. Future research must investigate the causal role of NF‐κB in IL‐17 receptor‐mediated pulmonary fibrosis.

IL‐17 has been shown to induce the expression of MMPs such as MMP‐2, MMP‐9, and MMP‐13 and is involved in myocardial remodeling. In cardiac fibroblasts and other myocardial cells, IL‐17 activates NF‐κB, C/EBP‐β, and AP‐1, leading to the production of chemokines, pro‐inflammatory cytokines, and adhesion molecules (Cortez et al., 2007). In systemic sclerosis (SSc), both IL‐1 and IL‐17 are elevated and influence the levels of IL‐6 and MMP‐9 in murine and skin fibroblasts (Park et al., 2018). These observations align with our findings demonstrating suppression of NF‐κB, levels of TNF‐α, IL‐6, and reduction in the expression of MMP‐2, MMP‐9 on neutralization of IL‐17RA and IL‐17RC in a murine model of BLM‐induced pulmonary fibrosis.

The fibrinolytic system has an important role in the progression of pulmonary fibrosis as it is involved in wound healing and repair. Animal models of pulmonary fibrosis, as well as IPF patients, have shown abnormal fibrinolytic activity (Crooks & Hart, 2015). During pulmonary fibrosis, PAI‐1 levels are increased, and this allows fibrin deposition, leading to the scarring of the lung (Huang et al., 2015). In this study, we investigated the effect of IL‐17RA and IL‐17RC neutralizing antibodies on the fibrinolytic system in the BLM‐induced pulmonary fibrosis model. Treatment with either IL‐17RA or IL‐17RC neutralizing antibody following BLM administration reduced the expression of PAI‐1 and increased the expression of uPA. Some markers were evaluated at certain time points due to their association with various stages of fibrotic development. Early‐stage markers such as uPA were examined on Day 14 to measure the initial process of fibrinolysis, while MMP‐9 levels were observed at Day 21 to determine ECM remodeling. Future research, especially studies conducted over time periods, will shed more light on this matter.

In summary, our data suggest that treatment with IL‐17RA or IL‐17RC neutralizing antibodies could alleviate pulmonary fibrosis by obstructing NF‐κB activation and excessive deposition of basement membrane proteins and alveolar thickening. Although our study highlights the therapeutic potential of neutralizing IL‐17A receptors, certain limitations remain. Only male mice were used in the study. Future studies including female mice are required to evaluate potential sex‐specific differences in IL‐17 receptor signaling and fibrosis progression. Treatment with IL‐17A receptor neutralizing antibodies decreased NF‐κB activation and cytokine expression, yet further experiments, including loss‐of‐function studies, could establish a causal link between neutralization of IL‐17A receptors and NF‐κB signaling. While intranasal delivery was effective in modulating fibrotic changes, direct evidence of bioavailability and lung tissue pharmacokinetic behavior has yet to be confirmed. The BLM‐induced pulmonary fibrosis model offers insights into the underlying fibrotic mechanisms, but it is acute and inflammatory‐driven, which may differ from the chronic progressive nature of IPF seen in humans. In addition to this, as IL‐17A and IL‐17F signal through a receptor complex consisting of IL‐17RA and IL‐17RC, the neutralization of IL‐17RA and IL‐17RC receptors may affect IL‐17F signaling. Further research, including knockout models, could help in studying the role of IL‐17A, IL‐17F, and their receptors in the progression of fibrosis and immune modulation.

Overall, the current study clearly indicates that the signaling of the IL‐17RA and IL‐17RC receptors plays a central role in the regulation of inflammatory and matrix remodeling processes in pulmonary fibrosis. Receptor‐selective inhibition was effective in modulating MMPs, restoring fibrinolysis equilibrium, and suppressing the development of pulmonary fibrosis.

## 5 CONCLUSION

This study demonstrates that IL‐17RA and IL‐17RC play important roles in ECM remodeling and fibrinolytic imbalance in BLM‐induced pulmonary fibrosis. The blockade of these receptors reduces inflammation, regulates MMPs, and restores the fibrinolytic balance. The statistical analysis using One‐way ANOVA followed by Sidak's multiple comparison test confirmed that treatment with IL‐17RA or IL‐17RC neutralizing antibodies reduced inflammation, ECM accumulation, and modulated the expression of MMPs, such as MMP‐2 and MMP‐9. Further, treatment with IL‐17RA or IL‐17RC neutralizing antibodies restored normal fibrinolytic activity by increasing uPA expression and decreasing PAI‐1 expression. These findings suggest treatment with either IL‐17RA or IL‐17RC neutralizing antibodies may restore fibrinolytic activity and modulate MMP expression, NF‐κB activation and ECM deposition, which are key processes in fibrosis across multiple organs, including heart, liver, and kidney. This suggests that blockade of IL‐17A receptors may have potential broader therapeutic relevance. However, the effects of IL‐17RA or IL‐17RC neutralizing antibodies may vary across different organs due to tissue‐specific interactions. Overall, this study highlights the potential of IL‐17RA and IL‐17RC as therapeutic targets in pulmonary fibrosis.

## AUTHOR CONTRIBUTIONS

**Yashodhar P. Bhandary:** Conceptualization; funding acquisition; supervision. **Rakshitha Charavu:** Conceptualization; data curation; formal analysis; investigation; methodology; project administration; software; validation; visualization. **Jeena Thrikkandiyoor Madambath:** Funding acquisition; investigation; methodology; project administration. **Akarsha B. Jain:** Methodology.

## FUNDING INFORMATION

This study is supported via funding from Yenepoya (deemed to be university) seed grant (YU/Seed grant‐131‐2022).

## CONFLICT OF INTEREST STATEMENT

The authors declare no conflict of interest.

## ETHICS STATEMENT

Scientific Review Board—YU/SRB/129/2022. Institutional Animal Ethics Committee—YU/IAEC/P12/2024.

## Supporting information


**Figure S1.** Western blot images.

## Data Availability

Data can be made available on request to the corresponding author.
