# Supplementary material for: IL‐17RA/RC blockade modulates the fibrinolytic system and MMP activity in bleomycin‐induced pulmonary fibrosis in male mice
Source: Physiol Rep. 2026 Jun 15;14(12):e70913. doi: 10.14814/phy2.70913 (PMC13269181; doi:10.14814/phy2.70913)
Supplement: Supplementary file 1 — Figure S1. Western blot images. [file PHY2-14-e70913-s001.docx]

**Supplementary Figure 1: Western blot images**

**Blots corresponding to Fig. 3**


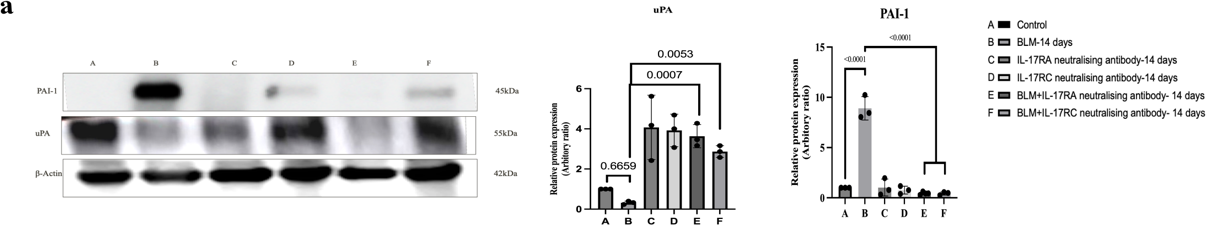


**Actin (42kDa)**

**
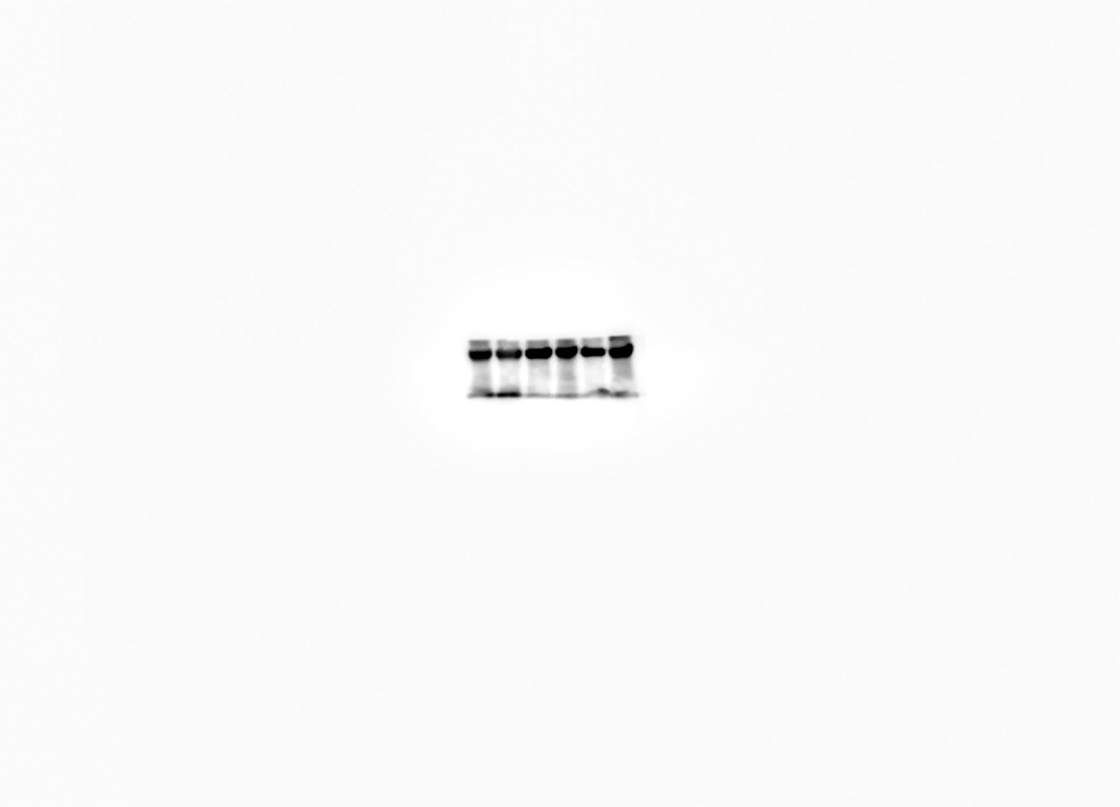
**

**uPA (55kDa)**


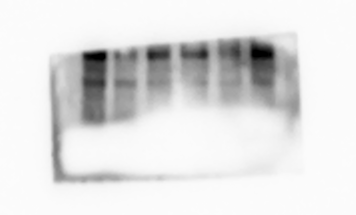
**
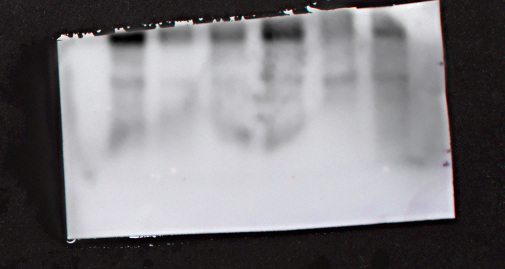
**

**PAI-1 (45kDa)**

**
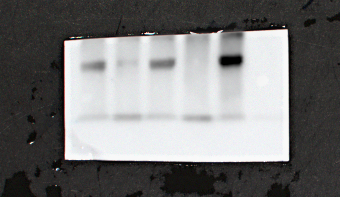
**

*Figure 3: Treatment with IL-17RA or IL-17RC neutralising antibodies restores the fibrinolytic system in BLM-induced lung fibrosis*

**Blots corresponding to Fig. 4**


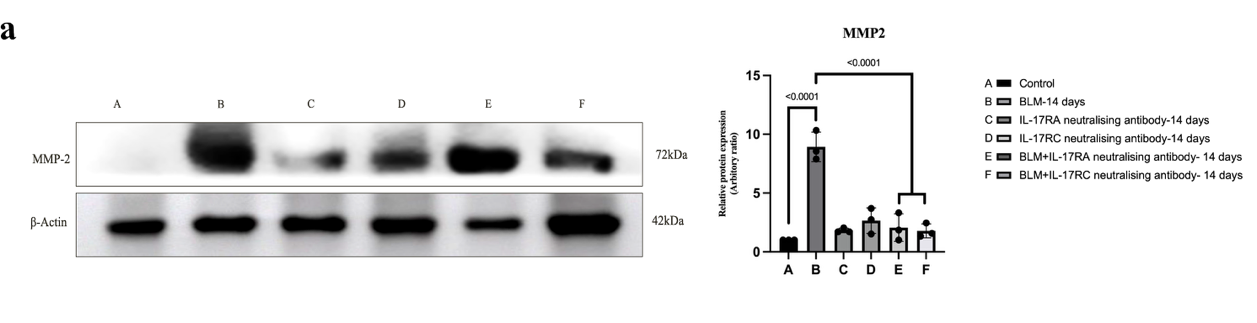


**Actin (42kDa)**

**MMP-2 (72kDa)**

**
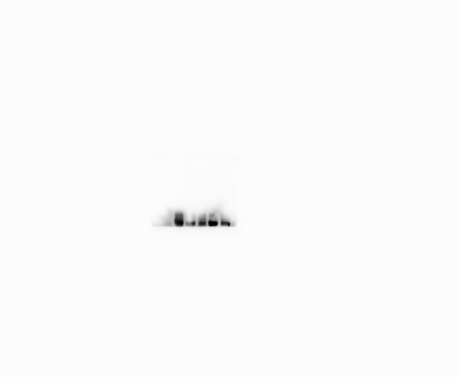
**

*Figure 4: Treatment with IL-17RA or IL-17RC neutralising antibodies reduces MMP-2 and MMP-9 expression and restores the fibrinolytic system in BLM-induced lung fibrosis*

**Blots corresponding to Fig. 5**


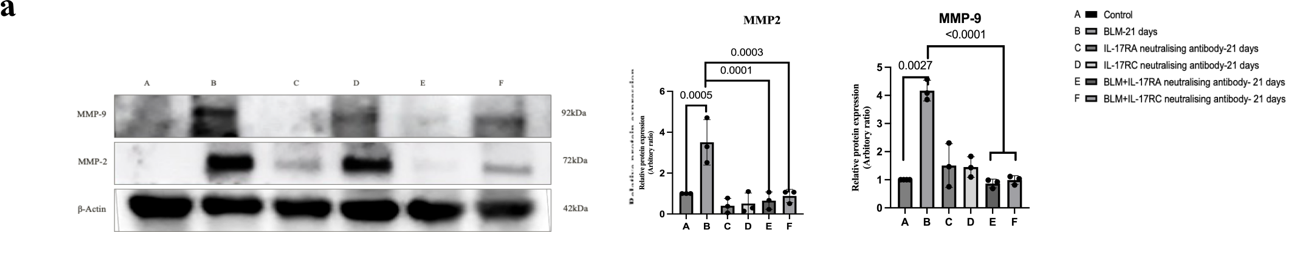


**Actin (42kDa)**

**MMP-2 (72kDa)**

**
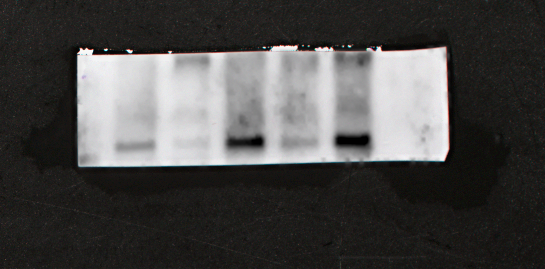
**

**MMP-9 (92kDa)**

**
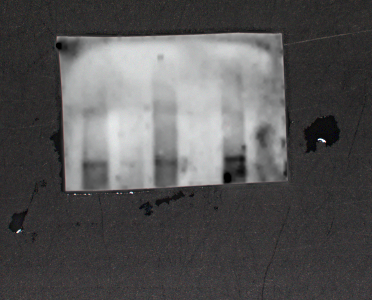
**

*Figure 5: Treatment with IL-17RA or IL-17RC neutralising antibodies reduces MMP-2 and MMP-9 expression and restores the fibrinolytic system in BLM-induced lung fibrosis*

**Blots corresponding to Fig. 6a: (14 days)**


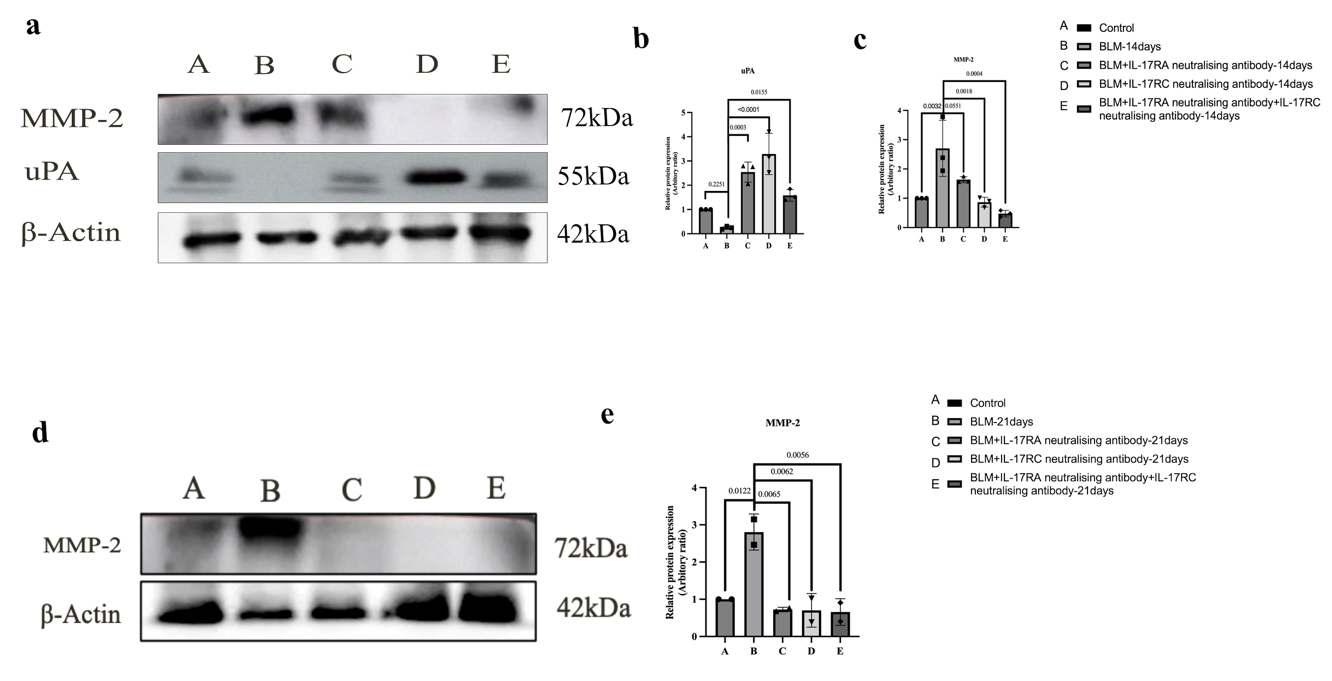


**Actin (42kDa)**


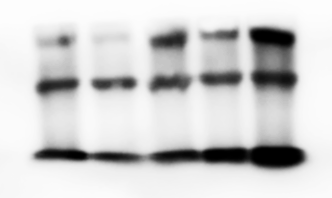


**uPA (55kDa)**

**MMP-2 (72kDa)**

**Blots corresponding to Fig. 6d: (21 days)**


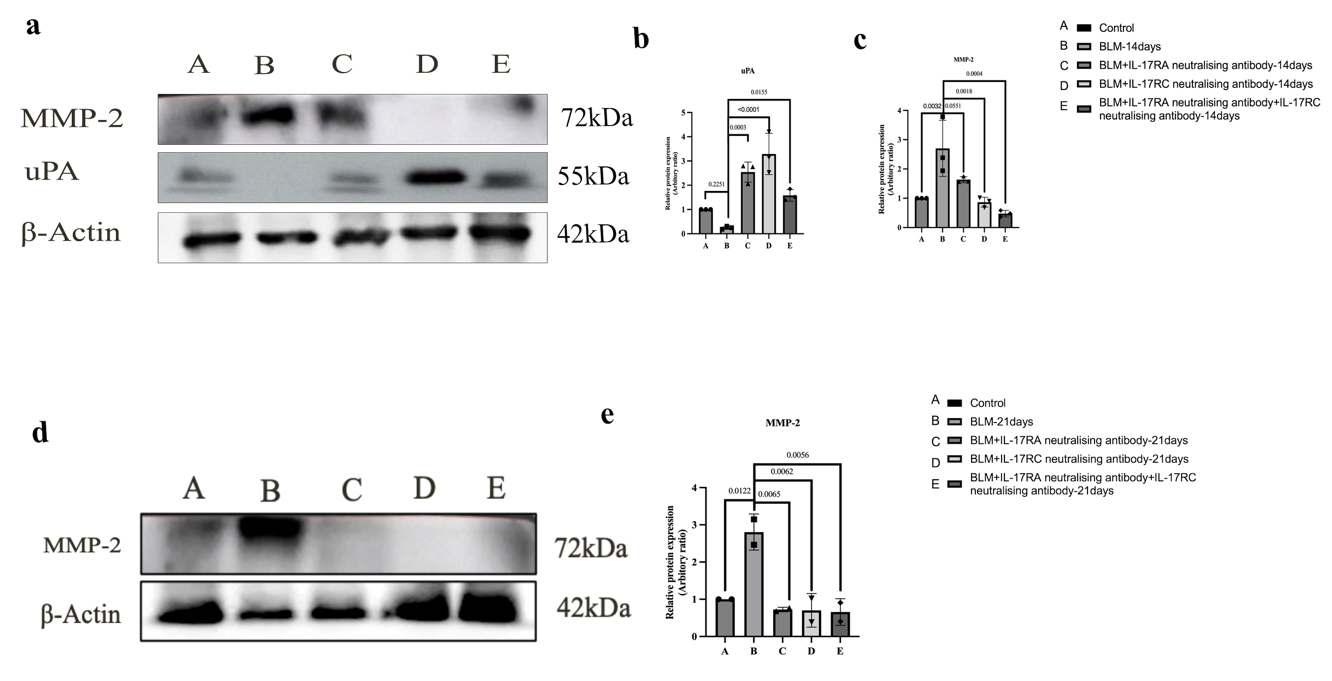


**Actin (42kDa)**


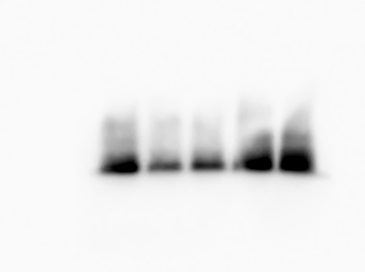


**MMP-2 (72kDa)**

**
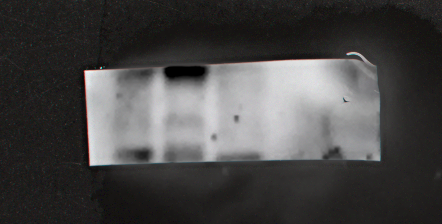
**

*Figure 6: Differential regulation of MMP-2 and uPA by IL-17A receptors*
